# Resilience, Social Participation Including Mutual Support, and Subjective Quality of Life Among Community-Dwelling Older Adults in Japan: A Cross-Sectional Study

**DOI:** 10.3390/healthcare14152320

**Published:** 2026-08-01

**Authors:** Junko Kazama, Fumiya Oohashi, Shinobu Kurabayashi, Yoshio Ohyama

**Affiliations:** 1 Independent Researcher, Maebashi 371-0804, Japan; kazajun2000@yahoo.co.jp; 2 School of Nursing, Ishikawa Prefectural Nursing University, Kahoku 929-1210, Japan; foohashi@ishikawa-nu.ac.jp; 3 Department of Nursing, Faculty of Health Care, Takasaki University of Health and Welfare, Takasaki 370-0033, Japan; kura@takasaki-u.ac.jp; 4 Graduate School of Health Sciences, Gunma University, Maebashi 371-8514, Japan

**Keywords:** community-dwelling older adults, quality of life, resilience, social participation, mutual support, healthy ageing, community nursing

## Abstract

**Highlights:**

**What are the main findings?**

- In this study sample, resilience was positively associated with subjective quality of life in community-dwelling older adults.
- In this study sample, older adults with higher levels of social participation involving mutual support had significantly higher resilience and subjective quality of life.

**What are the implications of the main findings?**

- Psychological resources such as resilience may contribute more strongly to subjective quality of life than social participation including mutual support.
- Community-based interventions should promote both resilience and social participation to support healthy ageing.

**Abstract:**

**Background:** Population ageing has increased the importance of maintaining the quality of life (QOL) of community-dwelling older adults. Resilience and social participation are considered important determinants of well-being in later life. However, little is known about how social participation involving mutual support relates to subjective QOL in Japanese community settings. This study examined associations between resilience and social participation, including mutual support and subjective QOL, among community-dwelling older adults. **Methods:** This cross-sectional survey was conducted in December 2021 among community-dwelling older adults in Japan. The study sample mainly comprised socially active older adults recruited through senior citizen clubs. Anonymous self-administered questionnaires were distributed to 434 individuals aged 65 years and older, and 258 valid responses were analysed. Subjective QOL was assessed using the Subjective QOL Scale, resilience using the Resilience Scale for Older Adults, and social participation, including mutual support, using a composite score based on neighbourhood relationships and participation in community activities. **Results:** Subjective QOL was positively correlated with resilience (ρ = 0.475, *p* < 0.001) and social participation including mutual support (r = 0.163, *p* = 0.009). After adjustment for self-rated health, the number of outpatient visits per month, and the number of daily medications, the association between subjective QOL and social participation including mutual support was no longer significant (pr = 0.104, *p* = 0.096), while resilience remained significantly associated with subjective QOL (pr = 0.417, *p* < 0.001). Participants with higher levels of social participation had significantly higher subjective QOL and resilience scores than those with lower levels of participation. **Conclusions:** In this study sample, resilience was more strongly associated with subjective QOL than social participation including mutual support. These findings highlight the importance of individualised community-based approaches that address both psychological resilience and opportunities for social participation when promoting well-being among older adults.

## 1. Introduction

The Health Japan 21 (the Third Term) initiative was launched in 2024 with the aim of extending healthy life expectancy and improving quality of life (QOL) in Japan. This national policy fosters not only improvements in individual health status but also health promotion through the development of supportive social environments, emphasising the importance of enhancing individual QOL [1]. Although no universal definition of QOL exists, the World Health Organization (WHO) defines it as individuals’ perceptions of their position in life within the context of their culture, value systems, goals, expectations, standards, and concerns, conceptualising it as a multidimensional construct that includes physical, psychological, and social dimensions [2]. Furthermore, population ageing and advances in medical care have resulted in an increasing number of older adults leading active and healthy lives despite chronic illnesses or disabilities. Therefore, maintaining subjective QOL, including the capacity to live positively and meaningfully, has become increasingly important among older adults [3].

Resilience is one of the key concepts contributing to the maintenance and improvement of QOL among older adults. It is defined as ‘the process and outcome of successfully adapting to difficult or challenging life experiences through mental, emotional, and behavioural flexibility’ and has attracted attention as the ability to maintain or recover a healthy state despite adversity [4,5]. Recent studies have demonstrated that resilience is an important protective factor associated with subjective well-being, QOL, and successful ageing among older adults [5,6,7]. Enhancing resilience in older adults has been shown to improve life satisfaction and QOL while also facilitating better social relationships and psychological well-being [6,7]. More recently, resilience has been conceptualised as an important psychosocial resource supporting subjective well-being and health-related QOL in later life [8].

In Japan, there exists an increasing need to effectively implement the community-based integrated care system, which aims to support older adults in continuing to live in their familiar communities, in response to the rapidly ageing society. To ensure its effective functioning, the expansion of the roles of ‘self-help’ and ‘mutual help’ within the framework of the ‘four types of support’ (self-help, mutual help, mutual public support, and public support) has been emphasised [9]. In this context, ‘mutual help’ refers to reciprocal and voluntary support relationships among community residents aimed at addressing community life issues [10]. Previous studies have suggested that promoting supportive neighbourhood relationships and community connectedness is important for strengthening mutual support within local communities [11]. Social participation and mutual support are interconnected in Japan because community activities involve reciprocal interactions and cooperative behaviour. In the present study, social participation and mutual support were operationalised as an integrated construct. However, urbanisation, shrinking family structures, and weakening neighbourhood ties have made it difficult to maintain traditional forms of mutual support in contemporary Japanese communities. Consequently, understanding how mutual support embedded in community participation contributes to the well-being of older adults is crucial.

Furthermore, ‘ageing in place’, defined as continuing to live in a familiar community, has gained attention as an important concept associated with subjective well-being and life satisfaction among older adults. Previous studies have suggested that older adults value remaining in familiar communities where social relationships, autonomy, and a sense of belonging can be maintained [12,13]. In addition, studies have suggested that social participation among older adults is associated with community belonging and resilience, indicating that individual psychological resources and community connectedness may jointly support participation in community life [14]. Moreover, social support and social connectedness within local communities may contribute to maintaining mental health and subjective QOL among older adults [15,16]. Such social participation provides opportunities to establish and maintain mutual support relationships through interactions and reciprocal assistance within the community, thereby supporting QOL in later life.

Previous studies have demonstrated that social participation is associated with better mental health, reduced frailty, and enhanced QOL among community-dwelling older adults [17,18]. However, social participation is not universally beneficial. It may also involve interpersonal stress and role-related burdens, suggesting that the quality and nature of participation should be considered when promoting social engagement among older adults [19]. Resilience may function as a psychological buffer that enables older adults to cope with interpersonal stress and role-related burdens associated with social participation, thereby helping to maintain subjective well-being and QOL. Although the benefits of social participation have been widely examined, few studies have focused on the potentially burdensome aspects of participation, particularly within the context of mutual support activities embedded in Japanese community settings. Furthermore, limited attention has been paid to how social participation involving mutual support relates to subjective QOL while considering the psychological and relational characteristics of older adults. Moreover, the influence of social participation on subjective QOL may differ according to older adults’ psychological resources, including resilience. Therefore, this study is significant in that it examines social participation from both positive and negative perspectives and explores its relationship with subjective QOL among community-dwelling older adults in the context of Japanese mutual-support-oriented communities.

Accordingly, the present study aims to clarify the associations of resilience and community-based activities involving mutual support with subjective QOL among community-dwelling older adults. It also examines the importance of resilience in maintaining and improving QOL and explores how social participation and mutual support relationships may influence subjective QOL among older adults living in the community through community activities.

## 2. Methods

### 2.1. Study Design

This study employed a cross-sectional design using anonymous self-administered questionnaires among community-dwelling older adults.

### 2.2. Operational Definition

Social participation including mutual support was operationally defined as activities based on interactions and reciprocal support among community residents. These activities included reciprocal support and cooperative actions aimed at addressing community life issues through resident organisations and neighbourhood relationships. They also included voluntary participation in hobby or sports activities, senior citizen clubs, and volunteer activities while maintaining connections with the local community. In Japanese communities, social participation and mutual support are closely intertwined because many community activities inherently involve reciprocal interactions and cooperative behaviours among residents. Therefore, social participation including mutual support was conceptualised and measured as an integrated construct in the present study. Because community participation in Japan often involves reciprocal interactions and mutual support, neighbourhood relationships and participation frequencies were integrated into a single exploratory composite index. The index was intended to capture the breadth and intensity of community engagement rather than represent a single latent construct. Therefore, internal consistency was not evaluated, and the score should be interpreted as a composite indicator of community participation involving mutual support.

### 2.3. Participants

Participants were community-dwelling older adults residing in City A, Japan. Eligible participants were individuals aged 65 years or older who were able to complete a self-administered questionnaire independently. Participants were recruited through senior citizen clubs in collaboration with the comprehensive community support centre in City A and through the researcher’s local neighbourhood association. Individuals who were unable to provide informed consent, unable to complete the questionnaire independently because of cognitive or physical difficulties, or returned questionnaires with substantial missing data were excluded from the analysis.

Based on Cohen’s criterion for a small-to-medium effect size (r = 0.20), a two-tailed alpha level of 0.05, and a statistical power of 0.80, a minimum sample size of 193 participants was estimated using G*Power version 3.1.9.7. The final sample size exceeded this requirement. No participants were excluded on the basis of sex, living arrangement, health status, or participation in community activities.

### 2.4. Survey Items

#### 2.4.1. Participants’ Characteristics and Community Activity Participation

Data collected on participants’ demographic characteristics and community activity participation included age group, sex, employment status, living arrangement, subjective health status, outpatient visits, medication use, awareness of contributing to the community, neighbourhood relationships, and frequency of participation in community activities.

#### 2.4.2. Social Participation Including Mutual Support Score

The items assessing social participation including mutual support were developed by the authors based on previous Japanese studies and community activity frameworks. The selected items were intended to capture both participation in community activities and the reciprocal interactions embedded in local community life. To assess social participation within the community, participation in ‘neighbourhood relationships’, ‘neighbourhood association activities’, ‘senior citizen club activities’, ‘volunteer activities’, ‘senior salons’, ‘circle activities’, and ‘ground golf’ was comprehensively integrated and quantified as the ‘social participation including mutual support score’. Each item was scored according to the frequency of participation or interaction, and a total score was calculated.

The questionnaire items and scoring system were as follows:

Degree of neighbourhood relationships:

- almost no interaction
- greeting only
- casual daily conversations
- mutual consultation and cooperation in daily life

Frequency of participation in community-based social activities:

- never
- once a year
- once every six months
- once a month
- two to four times a month
- five or more times a month

Because the score was designed to represent multiple forms of community participation and mutual support, it was regarded as an exploratory composite indicator rather than a unidimensional psychometric scale.

#### 2.4.3. Subjective QOL Scale

Subjective QOL was assessed using the scale developed by Yoshida and Yamazaki [3]. It comprises three items rated on a seven-point Likert scale and includes the following dimensions: ‘quality of life’ (living actively and vibrantly), ‘quality of daily living’ (living comfortably and pleasantly), and ‘quality of one’s life course’ (living a rich and happy life). Total scores range from 3 to 21, with higher scores indicating higher subjective QOL. The Cronbach’s alpha coefficient for this scale was 0.898, indicating good reliability.

#### 2.4.4. Resilience Scale for Older Adults

Resilience was assessed using the Resilience Scale for Older Adults developed by Ishimori et al. [20]. It comprises 21 items across the following seven subscales rated on a four-point Likert scale: ‘optimistic thinking and behaviour’, ‘sociability’, ‘situational analysis behaviour’, ‘friend and neighbourhood resources’, ‘positive engagement in life’, ‘family resources’, and ‘professional resources (physicians)’. Total scores range from 21 to 84, with higher scores indicating greater resilience. Cronbach’s alpha coefficients for all seven subscales exceeded 0.80, confirming adequate reliability.

### 2.5. Data Collection

Anonymous self-administered questionnaires were distributed in December 2021 to community-dwelling older adults in City A, Japan. Questionnaires were mailed to members of senior citizen clubs through the club office after obtaining cooperation from the comprehensive community support centre in City A. In addition, questionnaires were distributed to community residents through the researcher’s local neighbourhood association.

### 2.6. Data Analysis

Prior to analysis, the distributions of subjective QOL, resilience, and social participation including mutual support scores, were examined using the Shapiro–Wilk test for normality. Because all variables significantly deviated from a normal distribution, non-parametric statistical methods were employed for subsequent analyses. However, the skewness and kurtosis values indicated only minimal deviations from normality (Supplementary Table S1).

Descriptive statistics included frequencies, percentages, medians, and interquartile ranges (IQRs). Spearman’s rank correlation coefficients were calculated to examine associations among the study variables. In addition, partial Spearman correlation analyses were conducted to examine the associations between subjective QOL and resilience and between subjective QOL and social participation including mutual support, while controlling for subjective health status, the number of outpatient visits per month, and the number of daily medications. Partial Spearman correlations were estimated by rank-transforming all variables and then performing partial correlation analyses on the ranked data.

Participants were then divided into a low-score group (≤20 points) and a high-score group (≥21 points) based on the median score for social participation including mutual support. Mann–Whitney U tests were conducted to compare subjective QOL and resilience scores between these two groups. Effect sizes for the Mann–Whitney U tests were calculated using the standardised test statistic Z according to the formula: r = Z/√*n*. All analyses were conducted using IBM SPSS Statistics version 26, and the level of statistical significance was set at *p* < 0.05.

### 2.7. Ethical Considerations

Participation in this study was voluntary. The questionnaire package included a written explanation of the purpose and methods of the study as well as the handling of study results. Consent was considered to have been obtained when participants checked the consent box on the questionnaire and returned the completed questionnaire. All questionnaires were anonymous to ensure that participants could not be identified.

In addition, when recruiting participants through the researcher’s local community organisation, they were informed that participation was entirely voluntary and that refusal to participate would not result in any disadvantage, thereby minimising any potential coercion.

This study was approved by the Ethics Committee of Kiryu University (approved on 20 November 2021; Approval No. Kiryu 2021-01).

## 3. Results

### 3.1. Participant Characteristics

A total of 434 questionnaires were distributed, and 299 were returned (response rate: 68.9%). After excluding questionnaires with missing data, responses from 258 participants were included in the final analysis (valid response rate: 59.4%). Among the participants, 195 were men, and 63 were women. A total of 192 participants (74.4%) were aged in their 60s or 70s, whereas 66 participants (25.6%) were aged 80 years or older. Awareness of contributing to the local community was reported by 244 participants (94.6%) (Table 1).

### 3.2. Descriptive Statistics of Variables

The frequency distributions for the nine items categorised as ‘social participation including mutual support’ are shown in Table 2. They include neighbourhood relationships, neighbourhood association meetings, senior citizen clubs, volunteer activities at senior salons, other volunteer activities, welfare centres for older adults, senior salons, circle activities, and ground golf (Table 2).

Participants reported their most common neighbourhood relationships as ‘casual daily conversations’ (138 participants, 53.5%). When combined with those who reported ‘mutual consultation and cooperation in daily life’ (29.5%), approximately 80% of participants regularly interacted socially with neighbours. More than 70% attended neighbourhood association meetings on a somewhat regular basis, with ‘once a month’ being the most common response (28.3%). Participation in senior citizen clubs was reported by 77.1% of participants, with many engaging regularly, particularly ‘once a month’ (28.3%) and ‘two to four times a month’ (23.6%).

In contrast, participation rates in volunteer activities and senior salons were relatively low, with 67.4% and 67.8% reporting not having participated in volunteer activities at senior salons and not attending senior salons, respectively. Similarly, 57.8% reported no participation in other volunteer activities such as community cleaning or neighbourhood watch; however, among those who participate, ‘once a month’ (18.6%) was the most reported frequency.

Regarding hobby and sports-related activities, 43.4% of participants participated in circle activities, and 47.3% participated in ground golf. These activities tended to involve relatively frequent participation compared with other activities. The most reported frequency for circle activities and ground golf was ‘two to four times a month’ (22.9% and 21.3%, respectively), with some participating in ground golf even ‘five or more times a month’ (13.6%), indicating that a proportion of participants engaged in these activities at least weekly. Welfare centres for older adults had the lowest utilisation rate, with 81.0% reporting no participation. The study sample consisted predominantly of men (75.6%), which reflects the demographic characteristics of the senior citizen clubs and neighbourhood associations from which participants were recruited.

### 3.3. Summary Statistics for Subjective QOL and Resilience Scores

The median subjective QOL and resilience scores in this study were 17 (IQR: 15–19) and 64 (IQR: 57–70), respectively.

### 3.4. Correlation and Partial Correlation Analyses

An analysis of the associations between social participation including mutual support and other variables revealed statistically significant positive correlations with subjective QOL (ρ = 0.163, *p* < 0.01), resilience (ρ = 0.244, *p* < 0.01), and subjective health status (ρ = 0.167, *p* < 0.01). A significant positive correlation with the number of daily medications (ρ = 0.134, *p* < 0.05) was also observed. However, no significant associations were observed with household size or the number of outpatient visits per month (Table 3).

Partial Spearman correlation analyses that controlled for subjective health status, the number of outpatient visits per month, and the number of daily medications demonstrated that the positive association between subjective QOL and resilience remained statistically significant (pr = 0.417, *p* < 0.001). In contrast, the initially significant association between subjective QOL and social participation including mutual support (ρ = 0.163, *p* = 0.009) was attenuated after adjustment and was no longer statistically significant (pr = 0.104, *p* = 0.096) (Table 4).

To further explore the relationship between social participation including mutual support and resilience, associations with resilience subscales were examined. Social participation including mutual support was positively associated with the resilience subscales of positive engagement in daily life (ρ = 0.229, *p* < 0.001), sociability (ρ = 0.207, *p* = 0.001), and friend and neighbour resources (ρ = 0.173, *p* = 0.005) (Table 5). No significant associations were observed for positive thinking and behaviour, situational analysis behaviour, family resources, or professional resources.

### 3.5. Comparison of Subjective QOL and Resilience Scores Between Social Participation Groups

The summary statistics for social participation including mutual support score, revealed a score range of 9–38, with a median of 20 (IQR: 15–26). To examine differences in subjective QOL and resilience according to levels of social participation including mutual support, participants were divided into low-and high-score groups comprising 134 and 124 participants, respectively.

In the low-score group, the median subjective QOL and resilience scores were 17 (IQR: 14–18) and 62 (IQR: 56–69), respectively. In the high-score group, these values were 18 (IQR: 15–20) and 67 (IQR: 60–70), respectively.

Comparisons between the two groups revealed that subjective QOL scores (U = 9825.00, Z = 2.553, *p* = 0.011, r = 0.16) and resilience scores (U = 10,312.00, Z = 3.349, *p* = 0.001, r = 0.21) were significantly higher in the high-score group. However, although participants with higher levels of social participation including mutual support demonstrated significantly higher subjective QOL and resilience scores, these findings suggest that although statistically significant, the practical magnitude of the associations was limited (Table 6).

### 3.6. Comparison of Subjective QOL and Social Participation According to Resilience Level

Participants in the high-resilience group demonstrated significantly higher subjective QOL scores and social participation involving mutual support than those in the low-resilience group (Table 7).

## 4. Discussion

### 4.1. Main Study Findings

A key feature of this study was the simultaneous examination of resilience as an individual resource and social participation including mutual support as a community resource in relation to subjective QOL among community-dwelling older adults. The findings demonstrated that subjective QOL was positively associated with resilience, subjective health status, and social participation including mutual support. However, the partial correlation analysis controlling for health-related variables revealed that the association between social participation including mutual support and subjective QOL was not statistically significant. These findings suggest that subjective QOL among older adults may not be determined solely by the amount of participation in community activities but by the influence of health status and psychological resources.

Previous research has suggested that well-being and QOL in later life are influenced not only by physical health but also by social relationships and psychosocial resources, highlighting the multidimensional nature of healthy ageing [21,22]. The findings of the present study align with previous findings, further suggesting the importance of individualised support in the care of older adults.

The predominance of male participants may also have influenced the present findings. In Japan, retired men often experience difficulties establishing social relationships and participating in community activities after retirement. Resilience may therefore play an especially important role in facilitating adaptation to post-retirement life and maintaining subjective QOL among older men.

### 4.2. Association Between Resilience and Subjective QOL

In this study, a relatively strong positive association was observed between resilience and subjective QOL. Resilience has been conceptualised as a psychological resource that enables flexible adaptation to adversity and stressful situations and identified as an important factor supporting life satisfaction and successful ageing among older adults) [6,7]. More recent research has suggested that resilience is shaped not only by individual characteristics but also by social relationships and environmental resources, highlighting its multidimensional nature in later life [5,6,7].

In the present study, participants with higher levels of social participation including mutual support also demonstrated significantly higher resilience scores. This suggests that social interaction and opportunities to assume social roles through community activities may support psychological adaptability among older adults. Previous studies have also indicated that resilience and social support are closely associated with subjective well-being in later life [5].

### 4.3. Relationships Among Social Participation Including Mutual Support, Resilience, and Subjective QOL

In recent years, social participation among older adults has attracted attention as a crucial factor associated with the prevention of social isolation and the maintenance of psychological well-being [14]. In the present study, social participation including mutual support was positively associated with subjective QOL, suggesting that social interactions and roles obtained through community activities may support self-efficacy and provide meaning in life among older adults.

However, in the partial correlation analysis controlling for health status, the association between social participation including mutual support and subjective QOL was no longer statistically significant, suggesting that participation in community activities may not directly enhance QOL but may instead be indirectly associated through health status and psychological resources. Specifically, older adults with greater physical and psychological capacity may be more likely to participate in community activities, suggesting the possibility of a health selection bias in the present findings.

The present findings may also reflect the characteristics of the study sample, which comprised predominantly socially active older adults recruited through senior citizen clubs and neighbourhood associations. Most participants reported maintaining community relationships and feeling that they contributed to the local community. Therefore, the additional benefits of social participation including mutual support on subjective QOL, may have been attenuated because many participants already possessed substantial social resources and community connectedness. In contrast, social participation including mutual support may demonstrate a stronger association with subjective QOL among socially isolated or less active older adults who have fewer opportunities for social connection.

Social participation was particularly associated with positive engagement in daily life, sociability, and friend and neighbour resources, all of which were positively associated with subjective QOL. These findings suggest that community participation may contribute to subjective well-being by fostering behavioural and interpersonal dimensions of resilience rather than exerting direct effects alone. Furthermore, the present findings suggest that resilience may represent one potential pathway linking social participation including mutual support to subjective QOL, whereby community participation may promote behavioural and interpersonal resources that strengthen resilience and subsequently contribute to maintaining subjective well-being among older adults. Similar mediating mechanisms involving resilience have been reported among older adults in previous studies [23]. However, because the present study employed a cross-sectional design, this potential pathway should be interpreted cautiously and examined in future longitudinal studies.

Nevertheless, although the associations were statistically significant, the effect sizes were small. These findings indicate that although social participation including mutual support may be associated with subjective QOL and resilience among older adults, QOL is likely influenced by multiple factors, including physical health status, economic conditions, and psychological characteristics.

### 4.4. Need for Social Participation Support Considering Resilience Characteristics

This study suggested that among older adults with higher resilience, participation in community activities involving mutual support tended to be associated with subjective QOL. In contrast, among older adults with lower resilience, differences in subjective QOL according to levels of social participation were less apparent.

Because mutual support activities involve interpersonal interactions and social role performance, some older adults may perceive them as sources of interpersonal stress or psychological burden. Recent studies have also reported that the effects of social participation are influenced by individuals’ psychological states and adaptive capacities [24], highlighting the limitations of uniformly promoting social participation. Moreover, when social participation is accompanied by a sense of obligation, it may not necessarily contribute to improvements in QOL [19].

Therefore, support for older adults should not simply focus on increasing activity participation but should instead establish support systems that enable them to participate in community activities in ways that are manageable and appropriate to their individual values, psychological characteristics, and resilience profiles.

### 4.5. Implications for Community-Based Integrated Care and Community Nursing Practice

In recent years, the concept of ‘ageing in place’—whereby older adults continue living in familiar communities—has gained increasing international attention. Previous studies have suggested that remaining in familiar environments and maintaining community connectedness contribute to older adults’ well-being, autonomy, and sense of belonging [13,24].

In addition, social networks and reciprocal support relationships within communities have been reported to play important roles in preventing social isolation and maintaining health among older adults [15,17]. The findings of this study suggest that within Japan’s community-based integrated care system, support should address not only institutional resources but also individual resilience and reciprocal community relationships among older adults. Specifically, community nursing practice should involve comprehensive assessments that encompass levels of social participation, resilience, and psychological burden. Public health nurses and visiting nurses should support participation in community activities while considering older adults’ coping capacities and personal values, tailoring support to individual needs.

Because most participants were socially active and functionally independent older adults, ceiling effects may have attenuated the observed association between social participation and subjective QOL. Different findings may emerge among socially isolated older adults. In addition, social participation including mutual support was assessed using an exploratory composite index combining neighbourhood relationships and multiple forms of community participation. This approach was adopted because social participation and mutual support are closely intertwined in Japanese communities and often occur simultaneously through reciprocal interactions among residents. Nevertheless, the index has not been fully psychometrically validated. Therefore, the findings should be interpreted cautiously, and further studies are needed to examine its reliability and construct validity.

## 5. Limitations

The findings of this study were derived from a cross-sectional survey of mainly socially active older adults recruited through senior citizen clubs and neighbourhood associations. Therefore, the results cannot be generalised to all community-dwelling older adults. Moreover, the study sample was predominantly male, which further limits the generalisability of the findings. Given that older men and women may differ in their patterns of social participation, mutual support, and resilience, caution is warranted when applying these findings to broader populations of community-dwelling older adults.

Approximately three-quarters of the participants took part in senior citizen club activities, and most participants reported a sense of contributing to the local community. These characteristics suggest that the study population consisted predominantly of socially active older adults with relatively rich community resources. Consequently, selection bias may have occurred, potentially leading to an overestimation of the levels of social participation, resilience, and subjective QOL observed in this study. In addition, social participation including mutual support was assessed using an exploratory composite index that integrated neighbourhood relationships and multiple forms of community participation. Although this approach reflected the intertwined nature of social participation and mutual support in Japanese communities, the psychometric properties of the index have not been fully established.

Finally, the social participation including mutual support score was developed as an exploratory composite indicator of community participation instead of a psychometric scale. Therefore, its internal consistency was not examined. Future studies should further evaluate the validity and measurement properties of this composite indicator.

## 6. Conclusions

In this study sample, resilience showed a stronger association with subjective QOL than social participation including mutual support. These findings suggest that subjective well-being among older adults may be shaped by the interplay between individual psychological resources and community engagement. Community-based interventions should therefore promote both resilience and meaningful opportunities for social participation to support healthy ageing.

## Figures and Tables

**Table 1:** Participant Characteristics (*n* = 258).

Characteristic	*n* (%)
Age group	
60–79 years	192 (74.4)
≥80 years	66 (25.6)
Sex	
Male	195 (75.6)
Female	63 (24.4)
Employment status	
Employed	69 (26.7)
Not employed/Retired	189 (73.3)
Household size	
Living alone	21 (8.1)
Two persons	125 (48.4)
Three persons	57 (22.1)
Four or more persons	55 (21.3)
Self-rated health	
Poor/Fair	24 (9.3)
Average	112 (43.4)
Good/Very good	122 (47.3)
Outpatient visits per month	
None	58 (22.5)
Once	155 (60.1)
≥Two times	45 (17.4)
Daily medication use	
None	50 (19.4)
1–2 medications	95 (36.8)
3–5 medications	84 (32.6)
≥6 medications	29 (11.2)
Sense of contribution to the community	
Yes	244 (94.6)
No	14 (5.4)

**Table 2:** Frequency Distribution of Social Participation Involving Mutual Support (*n* = 258).

Activity		*n*	%
Neighborly relations	Barely interact/only exchange greetings	44	(17.0)
	Casual conversations on a daily basis	138	(53.5)
	To consult with each other and cooperate in daily life.	76	(29.5)
Neighborhood association meetings	Not participating	67	(26.0)
	About once/year	32	(12.4)
	About once/six months	51	(19.8)
	About once/month	73	(28.3)
	A few times/month	28	(10.9)
	≥5 times/month	7	(2.7)
Senior citizens’ clubs	Not participating	59	(22.9)
	About once/year	22	(8.5)
	About once/six months	21	(8.1)
	About once/month	73	(28.3)
	A few times/month	61	(23.6)
	≥5 times/month	22	(8.5)
Community salon volunteering	Not participating	174	(67.4)
	About once/year	9	(3.5)
	About once/six months	14	(5.4)
	About once/month	47	(18.2)
	A few times/month	11	(4.3)
	≥5 times/month	3	(1.2)
Other volunteer activities Ex. Park and shrine cleaning, Local senior citizens’ association events, Recyclable materials collection, School commute supervision, Local crime prevention committee member, Traffic safety officer, etc.	Not participating	149	(57.8)
	About once/year	7	(2.7)
	About once/six months	27	(10.5)
	About once/month	48	(18.6)
	A few times/month	19	(7.4)
	≥5 times/month	9	(3.1)
Senior welfare center activities	Not participating	209	(81.0)
	About once/year	19	(7.4)
	About once/six months	5	(1.9)
	About once/month	16	(6.2)
	A few times/month	8	(3.1)
	≥5 times/month	1	(0.4)
Community salons	Not participating	175	(67.8)
	About once/year	9	(3.5)
	About once/six months	11	(4.3)
	About once/month	51	(19.8)
	A few times/month	10	(3.9)
	≥5 times/month	2	(0.8)
Hobby/recreation circles	Not participating	146	(56.6)
	About once/year	5	(1.9)
	About once/six months	5	(1.9)
	About once/month	26	(10.1)
	A few times/month	59	(22.9)
	≥5 times/month	17	(6.6)
Ground golf	Not participating	136	(52.7)
	About once/year	7	(2.7)
	About once/six months	16	(6.2)
	About once/month	9	(3.5)
	A few times/month	55	(21.3)
	≥5 times/month	35	(13.6)

**Table 3:** Correlations among Study Variables (*n* = 258).

Variable	1	2	3	4	5	6	7
1. Subjective QOL	-						
2. Resilience	0.475 **	-					
3. Household size	0.071	0.071	-				
4. Self-rated health	0.399 **	0.281 **	0.029	-			
5. Number of outpatient visits per month	−0.104	0.017	−0.019	−0.312 **	-		
6. Number of daily medications	−0.079	0.12	−0.012	−0.249 **	0.673 **	-	
7. Social participation involving mutual support	0.163 **	0.244 **	−0.021	0.167 **	0.07	0.134 *	-

Note. Correlations were calculated using Spearman’s rank correlation coefficients. Social participation involving mutual support was assessed using a composite score derived from participation in neighborhood relationships, neighborhood association meetings, senior citizens’ clubs, community salon volunteering, other volunteer activities, senior welfare center activities, community salons, hobby/recreation circles, and ground golf. * *p* < 0.05, ** *p* < 0.01 (two-tailed).

**Table 4:** Partial Correlations between Subjective QOL and Key Variables (*n* = 258).

Variable	Partial Correlation Coefficient (pr)	*p*-Value
Subjective QOL and Resilience	0.417 ***	<0.001
Subjective QOL and Social Participation Involving Mutual Support	0.104	0.096

Note. Partial correlations were adjusted for self-rated health, number of outpatient visits per month, and number of daily medications. *p* < 0.001 ***.

**Table 5:** Associations between Social Participation Including Mutual Support and Resilience Subscales (*n* = 258).

Resilience Subscale	Spearman’s Correlation Coefficient (r)	*p*-Value
Optimistic thinking and behavior	0.070	0.263
Sociability	0.207 **	0.001
Proactive engagement in daily life	0.229 ***	<0.001
Situational analysis behavior	0.104	0.096
Neighborhood and friend resources	0.173 **	0.005
Family resources	0.051	0.417
Professional resources (physicians)	0.104	0.096

Note. Spearman’s rank correlation coefficients were calculated to examine the associations between social participation including mutual support and resilience subscales. *p* < 0.01 **, *p* < 0.001 ***.

**Table 6:** Comparison of Subjective QOL and Resilience between Low and High Social Participation Involving Mutual Support Groups (*n* = 258).

Variable	Low Group Median (IQR)	High Group Median (IQR)	U	*p*	r
Subjective QOL	17 (IQR: 14–18)	18 (IQR: 15–20)	9825.0	0.011	0.16
Resilience	62 (IQR: 56–69)	67 (IQR: 60–70)	10,312.0	0.001	0.21

Note. Participants were divided into low and high social participation involving mutual support groups based on the median score (20 points). Comparisons were performed using the Mann–Whitney U test. IQR = interquartile range.

**Table 7:** Comparison of Subjective QOL and Social Participation Including Mutual Support between Low and High Resilience Groups (*n* = 258).

Variable	Low Group Median (IQR)	High Group Median (IQR)	U	*p*	r
Subjective QOL	15 (IQR: 13–21)	18 (IQR: 17–21)	12,496.0	<0.001	0.44
Social Participation Involving Mutual Support	18 (IQR: 13–26)	22 (IQR: 16–26)	10,338.5	0.001	0.21

Note. Comparisons between groups were conducted using the Mann–Whitney U test. Participants were divided into low and high resilience groups based on the median resilience score. IQR = interquartile range.

## Data Availability

The datasets generated and/or analysed during the current study are not publicly available due to ethical and privacy restrictions but are available from the corresponding author upon reasonable request.

## Supplementary Materials

The following supporting information can be downloaded at: https://www.mdpi.com/article/10.3390/healthcare14152320/s1, Table S1. Distribution Characteristics and Normality Tests of the Study Variables.

## Abbreviations

The following abbreviations are used in this manuscript:

QOL	Quality of Life
WHO	World Health Organization
IQRs	Interquartile ranges
