# Supplementary material for: Resilience, Social Participation Including Mutual Support, and Subjective Quality of Life Among Community-Dwelling Older Adults in Japan: A Cross-Sectional Study
Source: Healthcare (Basel). 2026 Aug 1;14(15):2320. doi: 10.3390/healthcare14152320 (PMC13464666; doi:10.3390/healthcare14152320)
Supplement: Supplementary file 1 [file healthcare-14-02320-s001.zip › healthcare-4384962-supplementary.pdf]

Supplementary Table S1. Distribution Characteristics and Normality Tests of the Study Variables

Variable	Skewness	Kurtosis	Shapiro–Wilk W	p value
Subjective QOL	−0.708	0.327	0.938	<0.001
Resilience	−0.408	0.050	0.987	0.023
Social participation including mutual support	0.194	−0.847	0.970	<0.001

Note. Normality was assessed using the Shapiro–Wilk test. Although all variables showed statistically significant departures from normality, skewness and kurtosis values indicated only minimal deviations from a normal distribution. Therefore, all subsequent analyses were performed using non-parametric statistical methods.
